# Mutation screen and association studies for the fatty acid amide hydrolase (FAAH) gene and early onset and adult obesity

**DOI:** 10.1186/1471-2350-11-2

**Published:** 2010-01-01

**Authors:** Timo D Müller, Günter Brönner, Melanie Wandolski, Jophia Carrie, Trang T Nguyen, Brandon H Greene, André Scherag, Harald Grallert, Carla IG Vogel, Susann Scherag, Winfried Rief, Hans-Erich Wichmann, Thomas Illig, Helmut Schäfer, Johannes Hebebrand, Anke Hinney

**Affiliations:** 1 Department of Child and Adolescent Psychiatry and Psychotherapy, University of Duisburg-Essen, Essen, Germany; 2 Department of Psychiatry, University of Cincinnati, Genome Research Institute, Cincinnati, OH, USA; 3 Biocenter of the University of Wuerzburg, Wuerzburg, Germany; 4 Institute of Medical Biometry and Epidemiology, Philipps-University Marburg, Marburg, Germany; 5 Institute for Medical Informatics, Biometry and Epidemiology, University of Duisburg-Essen, Essen, Germany; 6 Helmholtz Zentrum Muenchen, Deutsches Forschungszentrum fuer Gesundheit und Umwelt, Neuherberg, Germany; 7 Department of Clinical Psychology and Psychotherapy, Philipps-University Marburg, Marburg, Germany

## Abstract

**Background:**

The orexigenic effects of cannabinoids are limited by activation of the endocannabinoid degrading enzyme fatty acid amide hydrolase (FAAH). The aim of this study was to analyse whether *FAAH *alleles are associated with early and late onset obesity.

**Methods:**

We initially assessed association of five single nucleotide polymorphisms (SNPs) in *FAAH *with early onset extreme obesity in up to 521 German obese children and both parents. SNPs with nominal p-values ≤ 0.1 were subsequently analysed in 235 independent German obesity families. SNPs associated with childhood obesity (p-values ≤ 0.05) were further analysed in 8,491 adult individuals of a population-based cohort (KORA) for association with adult obesity. One SNP was further analysed in 985 German obese adults and 588 normal and underweight controls. In parallel, we screened the *FAAH *coding region for novel sequence variants in 92 extremely obese children using single-stranded-conformation-polymorphism-analysis and denaturing HPLC and assessed the implication of the identified new variants for childhood obesity.

**Results:**

The trio analysis revealed some evidence for an association of three SNPs in *FAAH *(rs324420 rs324419 and rs873978) with childhood obesity (two-sided p-values between 0.06 and 0.10). Although analyses of these variants in 235 independent obesity families did not result in statistically significant effects (two-sided p-values between 0.14 and 0.75), the combined analysis of all 603 obesity families supported the idea of an association of two SNPs in *FAAH *(rs324420 and rs2295632) with early onset extreme obesity (p-values between 0.02 and 0.03). No association was, however, found between these variants and adult obesity. The mutation screen revealed four novel variants, which were not associated with early onset obesity (p > 0.05).

**Conclusions:**

As we observed some evidence for an association of the *FAAH *variants rs2295632 rs324420 with early onset but not adult obesity, we conclude that the *FAAH *variants analyzed here at least do not seem to play a major role in the etiology of obesity within our samples.

## Background

An emerging body of evidence indicates that the endogenous cannabinoid system is implicated in the regulation of food intake and body weight maintenance. The endocannabinoid system comprises the cannabinoid receptors CNR1 and CNR 2 the endocannabinoids as their endogenous ligands and the enzymes responsible for their biosynthesis and degradation [1]. The most prominent endocannabinoids are N-arachidonoylethanolamine (anandamide) [2] and 2-arachidonoylglycerol (2-AG) [3]. Both are implicated in the regulation of food intake as exogenous application of anandamide [4,5] and 2-AG [6] stimulate food intake through activation of CNR1. Additionally, hypothalamic levels of both, anandamide and 2-AG are increased in response to fasting and decline upon refeeding [6]. Furthermore, rodents with disturbed leptin signal transduction (*ob/ob*, *db/db *mice as well as *fa/fa *rats) show elevated levels of endocannabinoids in the hypothalamus and leptin treatment of *ob/ob *mice decreased hypothalamic levels of both anandamide and 2-AG [7].

A characteristic feature of endocannabinoids is that they are synthesized by cells on demand and undergo a rapid degradation through specific hydrolases and lipases [1,8,9]. Among these the fatty acid amide hydrolase (FAAH) figure prominently [10]. FAAH is a membrane-bound 60-65 kDa protein that is widely distributed throughout the periphery and the brain with specific central localization in the hypothalamus, hippocampus, brain stem, cerebral cortex and striatum [10,11]. Under alkaline conditions, FAAH rapidly inhibits the orexigenic effects of anandamide by degrading it to ethanolamine and arachidonic acid [1]. In accordance to its pivotal role in degradation of anandamide, *FAAH *mRNA expression is decreased by 59% in adipose tissue of obese women, as compared to lean controls, whereas circulating levels of anandamide and 2-AG were increased by 35% and 52% respectively [12]. Additionally, FAAH activity and expression is decreased in lymphocytes of obese leptin deficient *ob/ob *mice [13] and leptin mediated STAT3 activation activates a CRE-like binding site on the *FAAH *promoter [14]. Furthermore, association with obesity had recently been reported for the A/A genotype of the *FAAH *SNP rs324420 [15]. The SNP leads to the non-synonymous exchange from proline to threonine at position 129 of the FAAH protein (Pro129Thr). Functional *in-vitro *studies further revealed that the 129Thr variant decreased the expression and activity of FAAH in humans, thus seemingly corroborating the implication of the A-allele at rs324420 in human obesity [16]. However, other studies could not substantiate the association of rs324420 with obesity [17-19].

As FAAH counteracts the orexigenic effects of endocannabinoids through their rapid degradation, genetic variation in *FAAH *that leads to decreased enzyme activity and thus increased levels of endocannabinoids might be implicated in the etiology of obesity. The aim of this study was therefore to test for association of selected gene variants in *FAAH *with obesity.

## Methods

### Study subjects

The ascertainment strategy was previously described in detail [20]. The (extremely) obese children and adolescents had age and gender specific BMI percentiles above 90^th ^(70% above 99^th^). Written informed consent was given by all participants and in the case of minors, by their parents. The study was approved by the Ethics Committees of the Universities of Marburg and Duisburg-Essen and carried out according to the Declaration of Helsinki.

**The obesity trios **comprised 521 (233 male) German (extremely) obese children and adolescents (mean BMI of 31.86 ± 5.99 kg/m^2^, mean BMI percentile: 98.98 mean age 13.42 ± 3.09 years) and both biological parents. The parents had a mean BMI of 30.02 ± 6.38 kg/m^2 ^and a mean age of 42.32 ± 6.07 years.

**The 235 independent obesity families **included 501 (229 male) German (extremely) obese children and adolescents (235 obese index patients and at least one obese sibling) and both biological parents. The index patients had a mean BMI of 32.28 ± 5.91 kg/m^2 ^(mean BMI percentile 99.25 ± 1.48) and a mean age of 13.40 ± 2.63 years, the siblings had a mean BMI of 28.57 ± 5.29 kg/m^2 ^(mean BMI percentile 97.37 ± 2.85) and a mean age of 15.15 ± 5.09 years. The parents had a mean BMI of 31.07 ± 6.03 kg/m^2 ^and a mean age of 43.06 ± 5.81 years.

**The epidemiological cohort **comprised 8,491 (4,250 male) adult individuals of a German population-based study group [KORA S3-S4]. The KORA sample is a representative study group of the population in the city and region of Augsburg (Bavaria, Germany) [21]. The here reported 8,491 individuals had a mean age of 49.3 ± 27.12 years and a mean BMI of 27.12 ± 4.59 kg/m^2^.

**The case-control sample **comprised 985 (360 male) German obese adults (mean BMI: 36.04 ± 5.39 kg/m^2^; mean age: 46.31 ± 14.74 years) and 588 healthy normal and underweight controls (mean BMI: 19.34 ± 1.94 kg/m^2^; mean age: 25.28 ± 4.42 years). All individuals were independent from the KORA cohort und all cases had a BMI at or above 30 kg/m^2^. The use of lean adults who were never overweight or obese during childhood (assessed by interview, [22]) as control group reduces the chances of misclassification compared to the use of lean children as controls who might become overweight in adulthood [22].

The *FAAH *coding region was screened in 92 German extremely obese children and adolescents of the trio samples who contributed to the initially observed overtransmission of the rs2295632 G-allele (Table 1). The screened individuals had a mean BMI of 33.61 ± 7.21 kg/m^2 ^(mean BMI percentile 99.2 ± 1.88) and a mean age of 14.13 ± 3.08 years. Association of the identified novel variants with early onset extreme obesity was assessed in the 521 German obesity trios.

**Table 1:** Genotypes and TDT results of the analysed SNPs in *FAAH *in the obesity trios

SNP^1^	Position^2^	Location exchange	N^3^	Genotypes (frequency %)^4^	Allele frequ.%^5^	Transm. rate^6^	TDT p-value
				GG 243 (0.478)	G: 0.68		
		putative		GA 209 (0.412)			
rs932816	g.-272G/A	promoter	508	AA 56 (0.110)	A: 0.32	0.53 (A)	0.14
							
		Exon 3,		CC 248 (0.690)	C: 0.83		
				CA 101 (0.282)			
rs324420	c.385C/A	Pro129Thr	359	AA 10 (0.028)	A: 0.17	0.44 (A)	0.06
							
		Exon 7		GG 258 (0.730)	G: 0.85		
				GA 87 (0.247)			
rs324419	c.895A/G	Cys299Cys	353	AA 8 (0.023)	A: 0.15	0.44 (A)	0.08
							
				GG 351 (0.978)	G: 0.99		
				GA 8 (0.022)			
rs873978	IVS7-228G/A	Intron 7	359	AA 0 (0.000)	A: 0.01	0.88 (A)	0.10
							
				GG 214 (0.610)	G: 0.77		
				GT 113 (0.322)			
rs2295632	*45G/T	3' region	351	TT 24 (0.068)	T: 0.23	0.44 (T)	0.05

^1 ^all SNPs were tested for Hardy-Weinberg equilibrium (p ≥ 0.05); ^2 ^numbers are given according to Dunnen and Antonarakis 2001 Hum Genet 109:121-124 [33]; ^3 ^number of obesity trios; ^4 ^genotype frequencies in the index patients of the obesity trios; ^5 ^allele frequencies in the index patients of the obesity trios. These are very similar to the allele frequencies reported for the European population in the dbSNP database http://www.ncbi.nlm.nih.gov/SNP/; ^6 ^transmission rate of minor allele

### Molecular genetic methods

#### Genotyping

We initially assessed association of five SNPs in *FAAH *(rs324420, rs324419, rs873978, rs2295632 and rs932816) with early onset extreme obesity. SNP rs324420, rs324419, rs873978 and rs2295632 were genotyped in 368 German obesity trios using matrix-assisted laser desorption/ionization time-of-flight mass spectrometry (MALDI-TOF MS, Sequenom, San Diego, CA). The *FAAH *promoter SNP rs932816 was genotyped in 521 German obesity trios using restriction-fragment length polymorphism analyses (PCR-RFLP). SNPs with nominal TDT p-values ≤ 0.1 were subsequently analysed in additional 235 independent obesity families. The 8,491 adult individuals of the population-based KORA cohort were analyzed using MALDI-TOF mass spectrometry. Additionally, we genotyped the *FAAH *SNP rs324420 in 985 independent German obese adults and 588 normal or underweight controls using PCR-RFLP.

#### Mutation screening

Single stranded conformation polymorphism analysis (SSCP) and denaturing high pressure liquid chromatography (dHPLC) were performed 92 extremely obese children and adolescents which mainly contributed to the observed overtransmission of the rs2295632 G-allele (Table 1). The coding region of *FAAH *plus 79 nucleotides (nt) of the 5'-untranslated region (UTR) and 154nt of 3'-UTR was amplified from genomic DNA using PCRs to yield 17 fragments. The corresponding primer sequences, PCR conditions and restriction enzymes can be obtained from the authors. SSCP and dHPLC were performed as described previously [23]. All amplicons with SSCP or dHPLC patterns deviant from wild type patterns were re-sequenced as described [22]. The implication of the identified novel variants in *FAAH *for childhood obesity was subsequently assessed in 521 German obesity trios using PCR-RFLP or Taqman^® ^genotyping followed by TDT analyses [24].

#### TaqMan assay

the identified rare *FAAH *SNP rs41305628 was genotyped using TaqMan^® ^allelic discrimination assay (Applied Biosystems, Germany), call rates > 95%, with 100% concordance of duplicates, using a Custom Assay. For validity of the genotypes, alleles were rated independently by at least two experienced individuals. Discrepancies were resolved unambiguously either by reaching consensus or by retyping.

### Statistics

Using Haploview 4.0 we estimated that the analyzed five SNPs cover the common genetic variability of *FAAH *with a mean max r^2 ^of 0.48 (assuming a minor allele frequency cut-off of 5% to define common variation using the HapMap data for the European population), Tests for Hardy-Weinberg equilibrium were carried out using an exact test [25]. To identify the most promising SNPs for replication studies, we first performed association analyses for five SNPs in up to 521 obesity trios using the transmission-disequilibrium tests (TDT) [24]. SNPs with nominal p values ≤ 0.1 were subsequently analysed in additional 235 independent obesity families applying the pedigree disequilibrium test (PDT) [26]. We further considered both independent datasets in a combined analysis comprising the initial trios and the 235 obesity families using the PDT. For each SNP we also assessed multiplicative genotype relative risks (GRR) with a 95% confidence interval (95%-CI) based on the ideas of Cordell and Clayton [27] using the stata package 'gamenu' http://www-gene.cimr.cam.ac.uk/clayton/software/stata/ which leads to some minor differences regarding p-values when compared to the PDT results due to differences in estimation.

SNPs with nominal PDT p-values ≤ 0.05 in the combined analyses were consecutively analysed in the population-based KORA cohort regarding adult obesity. We stratified the KORA probands into obese cases (BMI ≥ 30 kg/m^2^, N = 1,858) and normal or underweight controls (BMI < 25 kg/m^2^, N = 2,818) and applied logistic regression using gender and age as covariates and coding genotypes under a log-additive genetic model. Assuming that the GRRs we estimated from the combined family sample are also observable in adults, we would have been able to confirm the association of these three variants with an estimated power between 80% and 97%. Sensitivity analyses assessing BMI quantitatively by linear regression in all KORA probands, generally supported our conclusions drawn below in the results section. The strongest BMI effect per allele was 0.07 kg/m^2 ^(p = 0.41) for SNP rs2295632. Note that a population-based sample of 8,491 individuals would have a statistical power ≥ 80% to detected much stronger BMI effects (about 0.2 kg/m^2^) only (given all other assumptions being similar to those in the nuclear families).

Association analysis for *FAAH *SNP rs324420 was also performed in an additional independent 985 obese cases and 588 lean and normal weight controls using the Cochran-Armitage exact trend test. This case-control sample has a power of about 60% to confirm the GRR effect observed in the initial combined family sample for rs324420.

All reported p-values are two-sided, nominal and not corrected for multiple testing. Power calculations were performed using the program QUANTO http://hydra.usc.edu/GxE applying a significance level of 5%, the observed multiplicative GRRs from the family sample (1.15-1.23) and the corresponding frequencies of the risk alleles (70% - 81%) under a (log-) additive genetic model.

## Results

No evidence for deviations from Hardy-Weinberg equilibrium was observed in all independent study groups (all exact p > 0.05). Analyses of five SNPs in *FAAH *in up to 521 German obesity trios revealed some evidence for an association of the G-allele at rs2295632 with early onset extreme obesity (transmission rate of the G-allele 0.56; nominal p = 0.045 Table 1). Moreover, we observed some evidence for transmission disequilibrium for three other *FAAH *SNPs (rs324420 rs324419 and rs873978) in the obesity trios (nominal two-sided p-values between 0.06 and 0.10 Table 1). Analyses of these four variants in additional 235 independent obesity families could not substantiate this observation (nominal two-sided p-values between 0.14 and 0.75 Table 2). However, as the small sample size of this group might have contributed to the observed lack of association, we subsequently considered both independent datasets jointly. In the combined analyses of the 603 obesity families we observed some evidence for an association of two SNPs in *FAAH *(rs324420 and rs2295632 r^2^:0.69) with childhood obesity (nominal two-sided p-values of 0.02 and 0.03 Table 2). Similarly, haplotype analysis for rs324420 and rs2295632 alleles supported this observation (omnibus two-sided nominal p = 0.0387 Table 3). However, in the population-based study we observed no evidence for an association of the two variants with adult obesity (all p ≥ 0.05; Table 4) or BMI (see Methods). Also analyses of rs324420 in 985 German obese adults and 588 normal and underweight controls revealed no association with adult obesity (nominal two-sided p = 0.85).

**Table 2:** Genotypes and PDT results of the independent analysis of 235 obesity families and combined analyses of a total of 603 obesity families

SNP^1^	N^3^	Genotypes (frequency %)^4^	Allele frequ. %^5^	Transm. rate^6^	PDT p-value for 235 independent families	PDT p-value and GRR with 95%-CI on 603 families^7^
rs324420	494	CC 320 (0.648)	C: 0.802	0.47 (A)	0.14	0.02 (1.23, 1.03-1.48)
		CA 152 (0.308)				
		AA 22 (0.044)	A: 0.198			
						
rs324419	491	GG 350 (0.713)	G: 0.838	0.51 (A)	0.75	0.18 (0.94, 0.77-1.15)
		GA 123 (0.251)				
		AA 18 (0.036)	A: 0.162			
						
rs873978	494	GG 485 (0.982)	G: 0.991	0.49 (A)	0.56	0.08 (2.50, 0.97-6.44)
		GA 9 (0.018)				
		AA 0 (0.000)	A: 0.001			
						
rs2295632	496	GG 266 (0.536)	G: 0.732	0.483 (T)	0.32	0.03 (1.15, 0.98-1.35)
		GT 194 (0.391)				
		TT 36 (0.073)	T: 0.268			

^1 ^all SNPs were tested for Hardy-Weinberg equilibrium (p > 0.05); ^2 ^numbers are given according to Dunnen and Antonarakis 2001 Hum Genet 109:121-124 [33]; ^3 ^number of obese children or adolescents of the 235 obesity families; ^4 ^genotype frequencies in the index patients of the 235 obesity families; ^5 ^allele frequencies in the obese children or adolescents of the 235 obesity families. These are very similar to the allele frequencies reported for the European population in the dbSNP database http://www.ncbi.nlm.nih.gov/SNP/; ^6 ^transmission rate of minor allele in 235 obesity families; ^7 ^p-value of the combined analyses of the 603 obesity families.

**Table 3:** Haplotype analysis of the *FAAH *SNPs rs324420 and rs2295632 in the 603 obesity families

rs324420	rs2295632	Frequency	Transmitted	Nontransmitted	Transmissionrate^1,2^
A	T	0.197	232	288	0.806
C	T	0.064	99	94	1.053
A	G	0.000	0	0	-
C	G	0.739	340	289	1.176

^1 ^Number of haplotypes were estimated by the EM-algorithm [34]; ^2 ^omnibus p-value for association is p = 0.0387

**Table 4:** Genotype frequencies and results of association analyses in the population-based KORA sample

SNP	BMI	Genotype frequency N (%)	Allele frequency (%)	OR^1^	95% CI	p-value^2^
rs2295632	< 25	CC 1575 (0.560)	C: 0.75	1.01	0.91 - 1.12	0.91
		CA 1068 (0.379)				
		AA 172 (0.061)	A: 0.25			
						
	≥ 30	CC 1051 (0.567)	C: 0.75			
		CA 685 (0.369)				
		AA 119 (0.064)	A: 0.25			
						
rs324420	< 25	CC 1876 (0.666)	C: 0.82	1.02	0.91 - 1.15	0.71
		CA 855 (0.303)				
		AA 87 (0.031)	A: 0.18			
						
	≥ 30	CC 1240 (0.668)	C: 0.81			
		CA 547 (0.294)				
		AA 71 (0.038)	A: 0.19			

^1 ^Odds ratios (OR) of the respective minor alleles were adjusted for sex and age; ^2 ^Association tests were performed applying generalized linear model with the log-transformed binomial link function; p-values are two-sided and not corrected for multiple testing.

In the mutation screen, we detected nine sequence variants, four of them are novel (Table 5). However, analyses of the rare (minor allele frequency < 0.1) variants (IVS1+22G/A, rs41305628 and IVS12-5C/T) in 521 German obesity trios revealed no evidence for association of these variants with early onset extreme obesity (all two-sided p ≥ 0.05).

**Table 5:** *FAAH: *results of a mutation screen in 92 German extremely obese children and adolescents

Gene	SNP	Position^1^	Location Exchange	Genotypes n (%)^2,4^	Allele frequency (%)^3,4^
*FAAH*		IVS1+22G/A	Intron 1	GG 31 (0.337)	G: 0.60
				GA 49 (0.533)	
				AA 12 (0.130)	A: 0.40
					
*FAAH*	rs324420	c.385C/A	Exon 3	CC 82 (0.901)	C: 0.95
				CA 09 (0.099)	
			Pro129Thr	AA 00 (0.000)	A: 0.05
					
*FAAH*		c.611C/T	Exon 5	CC 90 (0.989)	C: 0.99
				CT 01 (0.011)	
			Thr204Ile	TT 00 (0.000)	T: 0.01
					
*FAAH*		c.690C/G	Exon 5	CC 90 (0.989)	C: 0.99
				CG 01 (0.011)	
			Ser230Ser	GG 00 (0.000)	G: 0.01
					
*FAAH*	rs41305628	c.822G/A	Exon 6	GG 88 (0.957)	G: 0.98
				GA 04 (0.043)	
			Glu274Glu	AA 00 (0.000)	A: 0.02
					
*FAAH*	rs41309147	IVS6-41G/A	Intron 6	GG 77 (0.837)	G: 0.92
				GA 15 (0.163)	
				AA 00 (0.000)	A: 0.08
					
*FAAH*	rs324419	c.895C/T	Exon 7	CC 70 (0.761)	C: 0.88
				CT 22 (0.239)	
			Cys299Cys	TT 00 (0.000)	T: 0.12
					
*FAAH*		IVS12-5C/T	Intron 12	CC 79 (0.859)	C: 0.93
				CT 13 (0.141)	
				TT 00 (00.00)	T: 0.07
					
*FAAH*	rs2295632	IVS15+45G/T	3'UTR	GG 18 (0.196)	T: 0.60
				GT 74 (0.804)	
				TT 00 (00.00)	G: 0.40

^1 ^numbers are given according to Dunnen and Antonarakis 2001 Hum Genet 109:121-124 [33];^2 ^genotype frequencies in the 92 individuals used for the mutation screen; ^3 ^allele frequencies in the 92 individuals used for the mutation screen. ^4 ^As the mutation screen was performed in those individuals who contributed to the initially observed overtransmission of the *FAAH *SNP rs2295632 G-allele, this SNP is therefore not in Hardy-Weinberg-Equilibrium in these samples.

## Discussion

Analyses of five SNPs in *FAAH *in up to 521 German obesity trios and 235 independent obesity families revealed nominal evidence for association of two *FAAH *SNPs (rs324420 and rs2295632) with early onset extreme obesity. The *FAAH *SNP rs324420 A-allele has recently been reported to be associated with drug and alcohol abuse [28] and obesity [15]. Functional *in-vitro *studies further revealed that expression and activity of FAAH is reduced in isolated peripheral circulating T-lymphocytes of homozygotes for the rs324420 A-allele, thus seemingly underlining the observed association of rs324420 with obesity [16]. However, several other studies could not confirm the association of the rs324420 A-allele with obesity [17-19]. In this study we also report lack of association of the rs324420 A-allele with obesity. Instead, we observed some evidence for an association of the C-allele at rs324420 with childhood obesity. In light of these contradictory findings pertaining to the association of rs324420 alleles with obesity, it has to considered that associations of genetic variants with complex phenotypes may vary with age [29] and ascertainment scheme [30]. Accordingly, in the population-based study, we observed no association of this variant with adult obesity. Also analyses of rs324420 in 985 German obese adults and 588 normal weight controls did not provide evidence for an association. Due to discrepant results pertaining to the implication of SNP rs324420 with obesity, analyses of this variant in larger study groups are necessary to clarify whether this variant is implicated in the etiology of obesity. One of the limitations of our study is that the here analyzed variants cover the common genetic variability of *FAAH *with a mean max r^2 ^of 0.48. Thus, it is possible that other common variants which are not in LD with the analyzed variants, might be of greater importance in the etiology of childhood and/or adult obesity. However, although rather small our mutation analysis in 93 obese children and adolescents did not hint to a major impact of additional mutations in *FAAH *in weight regulation. Moreover, *FAAH *variants were not reported among the top-hits of recent meta-analyses of genome-wide association studies for BMI or obesity [31,32]; potentially also indicating their minor importance for the etiology of obesity.

## Conclusions

In summary we observed some evidence for an association of two *SNPs *in FAAH (rs324420 and rs2295632) with early onset but not adult obesity. This finding is in line with previous results showing lack of association of rs324420 with adult obesity in a large population-based study [17]. As our data were not corrected for multiple testing, we thus conclude that the *FAAH *variants analysed here may play a major role in the genetic etiology of obesity within our samples. To finally answer this question, further studies in larger sample preferentially child and adolescents with an extreme phenotype should be analysed.

## Competing interests

The authors declare that they have no competing interests.

## Authors' contributions

TDM helped to carry out the molecular genetic studies, participated in design and interpretation of data and drafted the manuscript. MW and JC carried out the mutation screen under supervision of GB. GB and SS also participated in the study design and interpretation of data. TTN and BG performed the statistical analysis and helped to draft the manuscript under supervision of AS and HS. HG carried out the molecular genetic studies using MALDI-TOF mass spectrometry under supervision of TI and H-EW. TI, H-EW and WR made substantial contributions to the acquisition of data and contributed to the conception, design and interpretation of data. JH and AH conceived the design and directed study coordination; helped to draft the manuscript and revised it critically. All authors read and approved the final manuscript.

## Pre-publication history

The pre-publication history for this paper can be accessed here:

http://www.biomedcentral.com/1471-2350/11/2/prepub
